# Assessment of the Effectiveness of Ventilation Controls in Managing Airborne and Surface Lead Levels at a Newly Commissioned Indoor Shooting Range

**DOI:** 10.3390/ijerph191811711

**Published:** 2022-09-16

**Authors:** Robert Alcock, Magdalena Wajrak, Jacques Oosthuizen

**Affiliations:** 1 School of Medical and Health Sciences, Edith Cowan University, Joondalup, WA 6027, Australia; 2 School of Science, Edith Cowan University, Joondalup, WA 6027, Australia

**Keywords:** lead, indoor, shooting range

## Abstract

Lead levels at a new indoor target shooting range were assessed using both personal and environmental air monitoring methods. Surface swabs were collected from representative locations throughout the range prior to, and at the conclusion of, shooting. Personal samples were compared against the current Australian Workplace Exposure Standards with all results exceeding statutory limits. Static environmental samples exceeded the Air National Environmental Protection Measures (NEPM) air quality standard. Surface swabs showed significant increases from pre- to post-shooting with levels exceeding recommended limits. The performance of the mechanical ventilation system was also assessed and airflow levels were below the required rate at all test locations when compared against the values recommended by the National Institute for Occupational Safety and Health (NIOSH). Users of the indoor shooting range are at risk of potential exposure to hazardous levels of lead. It was recommended the ventilation system be reviewed by a suitably qualified ventilation engineer to ensure it is operating within the required parameters and able to transport generated lead contaminant away from the shooters breathing zone. A thorough cleaning regime should be implemented by the club to minimise surface lead loadings throughout the facility.

## 1. Introduction

Although not as popular as in some countries, recreational shooting in Australia is a growing sport with approximately 640,000 participants nationally [1]. The health effects of lead are well-known and the potential adverse exposures within both the occupational and recreational shooting communities have been thoroughly documented. Previous studies have identified the blood lead levels of recreational shooters may be more than forty times higher than those of the general public [2].

Lead exposure has been associated with kidney damage, hypertension, cardiac disease and decreased fertility [3]. Lead is also classified as a probable human carcinogen and, depending on the length and level of exposure, may result in either acute or chronic toxicity [4]. If inhaled or ingested, lead is readily transported throughout the body where it may adversely affect the central nervous, cardiovascular, reproductive, renal, and hepatic systems [4]. Consequently, most governments around the world have invested significant resources into reducing environmental lead levels [5]. However, lead exposure remains a significant risk amongst the shooting community [6]. Most of the blood lead level measurements from shooters compiled in thirty-six separate studies reviewed by Laidlaw et al. [7] were found to be above the recommended health level.

The risk of lead exposure when using indoor firing ranges has been well-documented, most prominently within the law enforcement and military industries [8]. Several studies have also been undertaken which focused on recreational and sporting shooters and it was reported that the risk of lead poisoning among shooters may be similar to those occupationally exposed [9]. A review of the New York State Heavy Metals Registry which reports on the lead testing results of New York residents found over half of all individuals with elevated lead reported that their exposures were from target shooting with no known occupational exposure [9]. In a South African cross-sectional study, a cohort of 87 recreational shooters were compared to a control group of 31 archers. Shooters had significantly higher blood lead levels (BLL) when compared to the archers with 42.4% of shooters and 5.9% of archers returning a BLL ≥ 10 μg/dL (*p* < 0.001). Issues identified as lacking in the indoor shooting facilities included inadequate ventilation, low levels of awareness of the hazards associated with lead, poor housekeeping, and a lack of personal-hygiene facilities [3]. In a subsequent study, Mathee et al. (2017) reported on two workers employed at a shooting range. The workers both had elevated blood lead levels and displayed clinical symptoms of lead poisoning [10]. A Lithuanian study, in 2022, reported that 20 workers at an indoor shooting range displayed symptoms of chronic lead poisoning with BLL ranging from 5.64 μg/dL to 45.8 μg/dL. It was concluded that shooting-range employees are at risk of daily lead exposure and, even at low levels, the constant exposures lead to elevated BLL concentrations over time [10].

The predominant route of lead exposure for indoor-firing-range users is the inhalation of airborne lead particles resultant from the ignition of the primer material and shearing of lead particles from the projectile as it passes through the barrel [2]. A primer is typically comprised of up to 35% lead styphnate and lead peroxide that ignites when struck by the firing pin to provide propulsion for the projectile [7]. The dust and fume generated by the primer ignition, along with the lead particles sheared from the projectile, are ejected at high pressure from the gun barrel with most of the material travelling at right angles to the direction of firing [7]. The dispersion pattern results in the release of lead-containing fume and particulate in close proximity to the shooters breathing zone [7].

Ingestion is also a credible exposure pathway with the released lead particulates settling on horizontal surfaces and the clothes and hands of the shooter [7]. This is particularly prevalent where there is insufficient airflow to transport the lead particulate away from the shooter [11,12]. Surface samples collected from an indoor firing range in California as part of an investigation conducted by the National Institute for Occupational Safety and Health (NIOSH), Washington, D.C., USA in 2013 identified the presence of lead on all surfaces tested [8]. Personal air sampling conducted as part of the same study also identified elevated airborne lead levels with results of 54 µg/m^3^ for personnel conducting nightly range maintenance and 64 µg/m^3^ for personnel conducting weekly range cleaning activities [8]. The California Division of Occupational Safety and Health Permissible Exposure Limit for airborne lead is 50 µg/m^3^ [13].

Lead particulate is also created as the projectile strikes the backstop with the backstops design and construction significantly influencing the amount of projectile fragmentation, the energy with which it is created, and the amount contained within the target housing [8]. Housekeeping activities including sweeping of the range and the maintenance of target butts have shown significantly elevated personal air sampling results during a study conducted in Germany [5]. Air sampling conducted during recreational shooting at an indoor range returned results of 0.86 mg/m^3^ whilst shooting and 7.14 mg/m^3^ whilst cleaning the bullet trap [5]. The ceiling level for lead at a workplace in Germany was 0.1 mg/m^3^ at the time the study was conducted [5].

The hazards associated with indoor firing ranges are well-documented, with shooters potentially exposed to hazardous levels of lead due to insufficient ventilation, poor housekeeping practices and other issues [13]. Airborne particulate levels at shooting ranges have been found to be 100–1000 times higher than background concentrations whilst shooting is taking place [14]. A significant portion of the released particulate is known to be heavy metals, predominantly lead, which presents a significant inhalation risk [14].

This project was undertaken to assess the effectiveness of the ventilation system in controlling airborne and surface lead levels at a newly built indoor target-shooting range in West Australia. The facility is to be used for club practice and competitions and the club wished to verify if existing ventilation controls are adequately managing lead levels throughout the facility. Prior to the shooting conducted on the day of monitoring, the range had only been used for air-rifle practice (no lead); however, the club wishes to also utilise the range for rimfire rile practice and competitions, which require the firing of lead rounds. It was anticipated that this purpose-built facility would be compliant and that the ventilation system would adequately control lead exposure of recreational shooters.

## 2. Materials and Methods

The shooting range assessed was a new facility and, prior to the day of sampling, the club had only allowed air riles to be used; therefore, no prior lead contamination associated with rimfire shooting was present. A combination of personal and environmental air monitoring was conducted whilst the range was in use to assess club members’ lead exposures. In addition, surface swab samples were collected from identified key areas to determine how much lead was deposited. An evaluation of the facilities mechanical ventilation system was also undertaken to determine its effectiveness at removing generated airborne lead particles from the around the shooting line and club members’ breathing zones. Club members and visitors were invited to attend the range on Saturday the 27 November 2021 to participate in the project by firing lead rounds and assist with the collection of personal samples. The ECU Human Research Ethics Committee granted approval for the project to commence prior to data collection (2021-02858-ALCOCK). All data was de-identified.

Inhalable dust samples were collected in accordance with Australian Standard 3640-2009: Workplace atmospheres—Method for sampling and gravimetric determination of inhalable dust [15]. Participants were asked to wear an SKC sampling pump attached to a belt around the waist. The pumps were connected via tubing to an IOM sampler fitted with a 25 mm polycarbonate filter which was located in the breathing zone of the participants. The sample train was calibrated to a flow rate of 2 L/m. Sample filters were analysed for both total dust (gravimetric) and lead content [16]. All samples were collected over a single day whilst the indoor shooting range was in use. Club members from the West Australian Smallbore Rifle Association were invited to shoot rimfire (lead rounds) rifles for the first time at the indoor range during a series of practice sessions. Shooters were given a thirty-minute timed period to shoot 20 targets from either a prone position or seated at a shooting bench. At the completion of each session, shooters would collect their targets for scoring and replace the targets with a new one. Used targets were placed on the Range Officers’ table before being collected and taken by the scorer to another room within the clubhouse for scoring. Scored targets were then returned to the Range Officers’ table for shooters to collect. Throughout the course of the day, a total of seven 20 target rounds were completed. Shooting commenced at approximately 9:30 am and finished at approximately 3:15 pm. Club members stopped for approximately one hour during the day for lunch in the dining room located at the opposite end of the clubhouse.

Personal monitoring samples (n = 8) were worn by volunteer club members for an approximate six-hour period whilst they participated in practice shooting. Seven of the samplers were set up as static environmental monitors on stands approximately 1.5 m above the ground in strategic locations throughout the shooting range that were deemed to be likely to be contaminated due to positioning in relation to targets and firing line. Although there is no “safe” level of exposure to lead, various agencies do publish exposure guidelines [4]. Air-monitoring-sample results were evaluated against the current Safe Work Australia Workplace Exposure Standard [16] and the National Environment Protection Measure for Ambient Air Quality standard [17]. A summary of the airborne exposure values used for this study is provided in Table 1.

Surface swab samples were collected from areas throughout the shooting range that members are likely to interact with throughout the course of a typical visit. Particular attention was paid to surfaces members are likely to touch with their hands throughout the course of the day, as well as those areas of high foot traffic. Swab samples were collected at the start of the day prior to shooting commencing and then repeated at the end of the day following the completion of shooting. 

Surface samples were collected in accordance with Lead in Surface Wipe Samples–Method 9100, Issue 2, NIOSH Manual of Analytical Methods (NMAM), Fourth Edition, 15 May 1996 [18]. On flat surfaces, a 10 cm × 10 cm template was placed over the area to be sampled. On cylindrical surfaces, such as handrails and doorknobs, the template was used to denote a 10 cm length and the diameter noted to calculate the surface area. The area within the template was wiped with a “Ghost Wipe” sampling wipe. Firm pressure was applied using 3 to 4 vertical S-strokes; the wipe was then folded in and wiped with 3 to 4 horizontal S-strokes and repeated a second time using 3 to 4 vertical S-strokes [18]. A walk-through survey was conducted to identify and purposively select surfaces more likely to be contaminated—such as floor and countertop surfaces where firing takes place—in order to capture the expected highest levels of lead contamination.

Surface swab results were compared against those published in Australian Standard 4361.2-1998 Guide to lead paint management Part 2: Residential and commercial buildings, which provides acceptable clearance levels for surface dust lead loadings to be used when determining the effectiveness of lead remediation. These levels are not an occupational exposure standard and are set based on the health risk to the general population including children and those susceptible to illness [18,19]. A summary of the acceptable surface lead loading levels is provided in Table 2.

Observations were made regarding the design of the indoor shooting range including the number and positioning of local exhaust ventilation (LEV) capture points and heating, ventilation and air conditioning (HVAC) system design and layout. Air velocity measurements were collected using a calibrated TSI 9515 hot wire anemometer to assess the LEV systems performance in accordance with the Health and Safety Executive’s guidelines [20]. Required airflows for indoor shooting ranges have been documented by the National Institute for Occupational Safety and Health [11] and are summarized in Table 3.

Laboratory analysis of the airborne samples and surface wipes was performed by the School of Science Analytical Facility, Edith Cowan University by inductively coupled plasma optical emission spectrometry (ICP-OES) and Voltametric Analysis.

## 3. Results

### 3.1. Summary of Personal Monitoring Results

A summary of the personal exposure sampling results is provided in Table 4. A total of eight samples were collected over a 6-hour period from shooters in various lanes of the shooting range. As can be seen in Table 4, in some cases, monitors were worn by multiple shooters to ensure sufficient sample volume was collected and to accommodate the varying durations shooters spent at the range. 

### 3.2. Summary of Environmental Monitoring Results

A summary of the environmental monitoring sample results is provided in Table 5 below. A total of seven samples were collected over a 7-hour period

### 3.3. Summary of Surface Swab Results

A summary of the surface swab sample results collected over a surface area in cm^2^ is provided in Table 6 and Table S1. A total of 18 samples were collected on flat surfaces throughout the shooting range. Swab samples were collected from representative surfaces prior to shooting commencing at the start of the day. The post-shooting sampling process was then repeated the following day.

### 3.4. Ventilation Results

A summary of the airflow measurements is provided in Table 7. Airflow measurements were collected from representative locations throughout the shooting range whilst it was in use and before shooting commenced. Airflow measurements were collected while the shooting range was closed with the ventilation and extraction systems switched off and access doors open. The airflow measurements were then repeated whilst the shooting range was in its “live” state, meaning all access doors were closed and the ventilation and extraction systems were operating. Airflow measurements collected whilst the range was “live” were taken in between shooting rounds whilst shooters were changing targets.

## 4. Discussion

All eight personal monitoring samples returned a result above the current Safe Work Australia (SWA) Occupational Exposure Limit (OEL), with some exceeding the value by many magnitudes. Although the shooting range is not a workplace, and its members not employees, the SWA OEL was adopted for the project as a surrogate indicator for acceptable exposure levels amongst an otherwise fit and healthy adult population. The current SWA OEL for lead is 0.05 mg/m^3^ measured over an eight-hour working day. As shown in Table 4, shooter 4 had the highest personal lead exposure result of 0.178 mg/m^3^, more than three times higher than the OEL, and this shooter was located on the opposite end of the range (lane 12) from shooter 2 in lane 3 who had the lowest exposure of 0.078 mg/m^3^ (difference of 0.1 mg/m^3^). The personal air monitoring sample results are similar to those reported by Ochsmann et al. [5] following monitoring of recreational shooters at an indoor shooting range in Germany.

Club members and visitors to the indoor shooting range may include children, women of childbearing age and other at-risk members of the community. Accordingly, the environmental monitoring results were compared against the National Air Quality Standard for lead established by the National Environment Protection Council (NEPC) as part of the National Environment Protection Measure for Ambient Air Quality (Air NEPM). The Air NEPM measures provide target air quality standards to minimise the risk of adverse health impacts from exposure to pollution for communities [17]. The Air NEPM measure for lead is 0.5 µg/m^3^ averaged over 12 months, and so it cannot be used to accurately assess exposure over a few hours. This air quality standard also advises there should be no exceedances of the 0.5 limit; however, all seven sample results were found to be greater than the National Air Quality Standard. The lowest sample result was 13 µg/m^3^ collected from the north-east corner of the room adjacent to the targets and the highest result was 58 µg/m^3^ collected from the south-east corner of the room behind the target butts. However, as mentioned, these results should be interpreted in the correct context. Shooters will not have this level of exposure if it were averaged over a 12-month period. The environmental sample results identified airborne lead concentrations similar to those found by the National Institute of Occupational Health Safety as part of an investigation conducted at an indoor firing range in California [8].

Surface swab sample results were compared against guideline values published in Australian Standard 4361.2-1998 Guide to lead paint management Part 2: Residential and commercial buildings, which provides acceptable clearance levels for surface dust lead loadings following lead remediation. These levels are not an occupational exposure standard and are set based on the health risk to the general population including children and those susceptible to illness [18].

Of the nine samples collected prior to shooting commencing, six results were found to be greater than the applicable guideline value. Sample locations included shooting benches, the floor in front of the firing line, and target butts. It should be noted the shooting benches are routinely used at the outdoor range and were moved into the indoor range for use on the day of monitoring which likely explains the presence of lead. It is assumed the presence of lead on the floor and target butts is a result of air rifles being fired at the indoor range previously. The only surfaces at which an acceptable lead loading was identified were the target faces and Range Officers’ table positioned behind the shooting line. 

In all cases, surface lead levels were significantly higher at the completion of shooting with swabs collected post range use showing an increase in lead levels throughout the entire range. The greatest increase in surface lead loading was identified on the shooting benches and floor in front of the firing line. All surface swab samples collected post shooting were higher than the applicable guideline value. During the day people frequently exited the range and attended other areas within the clubhouse such as the scoring room, bathroom and dining room without undergoing any personal decontamination or cleaning process. It is possible lead contamination is transferred on the hands, clothes and shoes of members as they move about the club’s facilities. It is also expected that higher risk surfaces such as used targets provide a significant potential contamination source when being moved between rooms in the clubhouse. 

**Figure 1:**
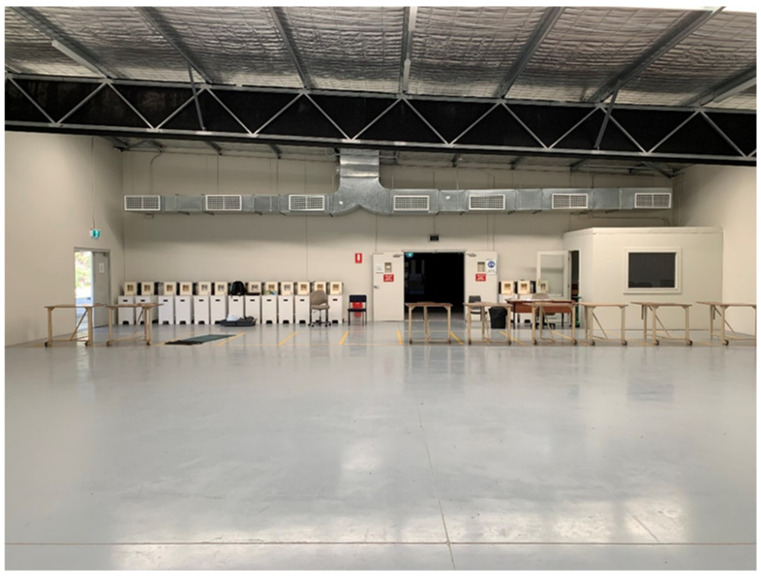
Supply air ducting and outlets.

**Figure 2:**
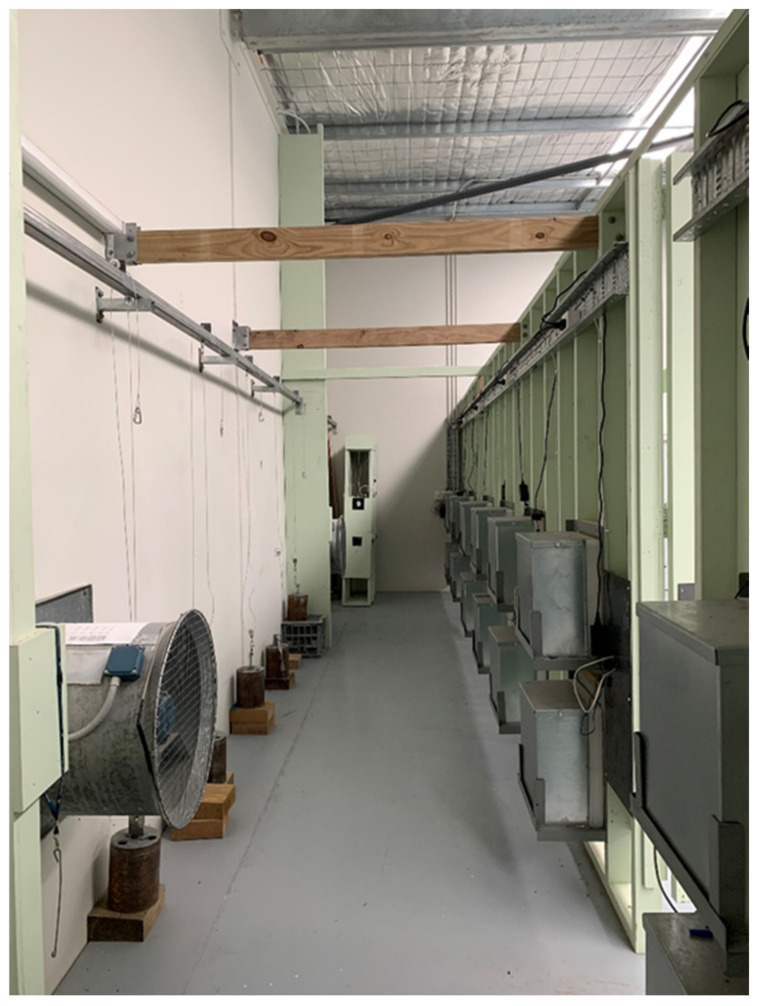
Mechanical extraction fans.

The shooting range has a mechanical supply air system and separate mechanical extraction systems which is required to be operating whilst the range is in use. The supply air system draws air from the roof of the building and is distributed via supply air ducting and grills along the upper western wall located behind the shooters as shown in Figure 1. The mechanical extraction system is comprised of three separate fans located behind the target butts on the eastern wall of the shooting range at ground level (Figure 2). The ventilation system has been designed to work with all access doors closed and the airflow moving in an east west direction away from the shooting line and towards the targets.

All airflow measurements were below the flow rates recommended by NIOSH for indoor firing ranges and showed variations in airspeed suggesting the supply air is not being evenly distributed [11]. The ventilation system is the principal means for controlling airborne lead levels throughout the shooting range and reducing the risks of inhalation, the primary exposure route [2]. The airflow direction and speed should be sufficient to transport the lead and other contaminants generated during firing away from the shooter’s breathing zone.

Given the poor performance of the ventilation system, these findings of lead contamination are not unexpected and there have been several studies conducted around the world where elevated lead levels have been detected not only in the air or on surfaces but also in the blood of recreational shooters and people employed in indoor shooting ranges. This is clearly an on-going problem globally [1,2,3,5,6,7,8,9,10,13,14]. 

The importance of adequate ventilation has been highlighted in many previous studies, with mechanical ventilation identified as the primary control for lead exposures in indoor shooting ranges [4]. Given the measured airflows in this study were significantly below the recommended values, the sampling results were not surprising and there is clearly a design or operational issue that will need to be addressed; this finding is particularly concerning as the study was conducted in a newly built, purpose designed facility. A suitably qualified ventilation engineer needs to be engaged to evaluate the ventilation system in its entirety and provide feedback on the design of the system, operating parameters, and required changes to ensure the proper operation of the system. It is expected improvements to the ventilation system will reduce airborne lead levels throughout the range, however, this needs to be verified through further monitoring following any changes made to the ventilation system. In addition to improving the ventilation, it is recommended the club undertake frequent cleaning of the range and all associated equipment to ensure surface lead levels are being maintained as low as practicable. Previous studies have shown cleanliness and housekeeping plays a significant role in shooters’ lead exposure [4]. Vacuum cleaners fitted with high efficiency particle air filters (HEPA) and wet cleaning methods are recommended to reduce possible exposures during cleaning activities [12]. Measures for limiting the possible transfer of lead contamination from the range to other “clean” areas within the building such as meeting and dining rooms should also be investigated and may include the use of sticky floor mats at exit doors. Members and visitors to the club should be instructed on the importance of thorough handwashing prior to eating and drinking and no food or drink should be consumed within the shooting range. Induction for new members should include information on lead hazards and how shooters can reduce their lead exposures; this should include instruction on the importance of thorough handwashing prior to eating and drinking [14].

## 5. Conclusions

This was a relatively small cross-sectional study; however, it was conducted in a new facility and the finding of extensive lead contamination after one day of shooting is of concern. Air monitoring results showed users of the indoor shooting range are at risk of exposure to elevated levels of lead whilst firing rimfire ammunition. All the personal monitoring sample results were above the current workplace exposure limit with some exceeding this limit by more than three times. Similarly, all the environmental air monitoring results were found to be higher than the recommended Air NEPM standard with elevated airborne lead levels identified throughout the shooting range. Surface swab results showed a significant increase in lead loading across all sampled surfaces with all samples collected at the conclusion of shooting returning a result above the guideline values published in Australian Standard 4361.2-1998 Guide to lead paint management Part 2: Residential and commercial buildings. The surface swab results also identified the presence throughout the range of elevated lead loading on surfaces users are expected to frequently interact with. 

Shooting ranges remain a credible lead exposure risk for their users globally, with deficiencies in ventilation and housekeeping likely to result in continuing exposures to hazards levels of lead. It is imperative that regulatory authorities should investigate this source of lead exposure and develop model regulations and guidelines that recreational and occupational (Armed forces and police) can implement as best practice controls. Specific design requirements need to be established that will ensure optimal ventilation and other engineering controls. The approach to reducing lead exposure should also be multi-faceted and should include proper training and inductions for both staff and club members and regular biological monitoring, particularly for staff, is recommended. Cleaning protocols also need to be developed that are facility specific with duties and responsibilities properly assigned to individuals [21]. The designers and manufacturers of guns and ammunition should also conduct research to develop alternative ammunition that does not contain lead, as the most effective control, elimination, does not appear to be a high priority amongst the shooting community.

## Figures and Tables

**Table 1:** Summary of Occupational Exposure Limits and Environmental Guideline Values.

Source	Description	Standard Name	Type	Value	Unit
Government of Western Australian	Occupational Exposure Limit	Mines Safety and Inspection Regulations 1995-Inhalable dust	TWA	10	mg/m^3^
Safe Work Australia	Occupational Exposure Limit	Lead, inorganic dusts & fumes (as Pb)	TWA	0.05	mg/m^3^
Air NEPM	National Air Quality Standard	Lead	1 year average	0.5	µg/m^3^

TWA = time weighted average.

**Table 2:** Acceptable surface dust lead loadings [18].

Location	Lead Loading	Unit
Interior floors	0.1	µg/cm^2^
Interior windowsills	0.5	µg/cm^2^
Exterior Surfaces	0.8	µg/cm^2^

**Table 3:** Recommended average air velocity [11].

Location	Airflow	Unit
Firing line	0.381	m/s
Minimum—all other locations	0.254	m/s

**Table 4:** Summary of personal-exposure monitoring sample results.

Sample Date	Sample Number	Shooter Number	Description of Shooter Activity	Time On	Time Off	Inhalable Dust (mg/m^3^)	Inhalable Dust OEL (mg/m^3^)	Lead (mg/m^3^)	Lead OEL (mg/m^3^)
27/11/2021	1	1	Lane 6—Bench	09:16	15:09	0.206	10	0.081	0.05
27/11/2021	2	2	Lane 3—Bench	09:15	15:05	0.205	10	0.078	0.05
27/11/2021	3	3	Lane 12—Bench	09:17	15:03	0.294	10	0.163	0.05
27/11/2021	4	4	Lane 12—Bench	09:17	15:03	0.465	10	0.178	0.05
27/11/2021	5	5	Lane 9—Prone	9:24	11:28	0.265	10	0.119	0.05
		6	Lane 7—Bench	11:29	12:02				
		7	Lane 2—Bench	13:15	14:00				
		8	Lane 7—Bench	14:04	15:07				
27/11/2021	6	9	Lane 11—Bench	09:20	15:10	0.376	10	0.158	0.05
27/11/2021	7	10	Lane 5—Bench	9:21	13:56	0.234	10	0.083	0.05
		11	Lane 8—Bench	14:03	15:14				
27/11/2021	8	12	Lane 2—Bench	9:23	11:26	0.153	10	0.082	0.05
		13	Range Officer	11:26	15:08				

**Table 5:** Summary of Environmental Monitoring Sample Results.

Sample Date	Sample Number	Location Description	Time On	Time Off	Inhalable Dust (mg/m^3^)	Inhalable Dust OEL (mg/m^3^)	Lead (µg /m^3^)	Air NEPM (µg /m^3^)
27/11/2021	9	South west corner of room adjacent shooting line	08:05	15:18	0.017	10	40	0.5
27/11/2021	10	North west corner of room adjacent shooting line	08:06	15:18	0.023	10	22	0.5
27/11/2021	11	South east corner of room adjacent targets	08:08	15:16	0.012	10	38	0.5
27/11/2021	12	North east corner of room adjacent targets	08:10	15:16	0.035	10	13	0.5
27/11/2021	13	Rear of room behind range officers desk	08:12	15:16	0.024	10	39	0.5
27/11/2021	14	South east corner of room behind target butts	05:15	15:20	0.012	10	58	0.5
27/11/2021	15	North east corner of room behind target butts	08:16	15:21	0.035	10	23	0.5

**Table 6:** Summary of Surface Swab Sample Results.

Sample Date	Sample Number	Location Description	Sample Area (cm^2^)	Lead Concentration (µg/cm^2^)	Acceptable Lead Loading	Comments
27/11/2021	SW01	Range Officers Table—Centre of table	100	0.370	0.5	Pre
27/11/2021	SW02	Shooting Bench Lane 2—Centre of bench	100	1.043	0.5	Pre
27/11/2021	SW03	Shooting Bench Lane 5—Centre of bench	100	2.110	0.5	Pre
27/11/2021	SW04	Shooting Lane 7— Floor in front of firing line	100	0.303	0.1	Pre
27/11/2021	SW05	Shooting Lane 9— Floor in front of firing line	100	0.557	0.1	Pre
27/11/2021	SW06	Target Face 3—Upper target	100	0.020	0.5	Pre
27/11/2021	SW07	Target Face 8—Lower target	100	0.026	0.5	Pre
27/11/2021	SW08	Shooting Lane 4— Top of upper target butt	100	1.019	0.5	Pre
27/11/2021	SW09	Shooting Lane 9— Top of lower target butt	100	0.618	0.5	Pre
27/11/2021	SW10	Range Officers Table—Centre of table	100	1.413	0.5	Post
27/11/2021	SW11	Shooting Bench Lane 2—Centre of bench	100	8.984	0.5	Post
27/11/2021	SW12	Shooting Bench Lane 11— Centre of bench	100	9.444	0.5	Post
27/11/2021	SW13	Shooting Lane 7— Floor in front of firing line	100	38.377	0.1	Post
27/11/2021	SW14	Shooting Lane 9— Floor in front of firing line	100	19.588	0.1	Post
27/11/2021	SW15	Target Face 3—Upper target	100	0.501	0.5	Post
27/11/2021	SW16	Target Face 9—Lower target	100	1.320	0.5	Post
27/11/2021	SW17	Shooting Lane 4— Top of upper target butt	100	1.502	0.5	Post
27/11/2021	SW18	Shooting Lane 9— Top of lower target butt	100	2.408	0.5	Post

**Table 7:** Summary of airflow measurements.

Sample Date	Measurement Number	Location Description	Airflow Range Live (m/s)	Airflow Range Closed (m/s)	Required Airflow (m/s)
27/11/2021	1	Shooting line, northern side of room	0.00	0.00	0.381
27/11/2021	2	Shooting line, center of range	0.01	0.00	0.381
27/11/2021	3	Shooting line, Southern side of room	0.12	0.01	0.381
27/11/2021	4	Centre of range, northern side of room	0.01	0.00	0.254
27/11/2021	5	Centre of range, center of room	0.01	0.01	0.254
27/11/2021	6	Centre of range, southern side of room	0.06	0.00	0.254
27/11/2021	7	Adjacent targets, northern side of room	0.01	0.00	0.254
27/11/2021	8	Adjacent targets, center of range	0.01	0.00	0.254
27/11/2021	9	Adjacent targets, Southern side of room	0.04	0.00	0.254
27/11/2021	10	Behind target butts, northern side of room	0.02	0.00	0.254
27/11/2021	11	Behind target butts, center of range	0.02	0.00	0.254
27/11/2021	12	Behind target butts, Southern side of room	0.06	0.00	0.254

## Data Availability

Not applicable.

## Supplementary Materials

The following supporting information can be downloaded at: https://www.mdpi.com/article/10.3390/ijerph191811711/s1, Table S1: Summary of Surface Swab Sample Results (pre and post-shooting).
