# Supplementary material for: Assessment of the Effectiveness of Ventilation Controls in Managing Airborne and Surface Lead Levels at a Newly Commissioned Indoor Shooting Range
Source: Int J Environ Res Public Health. 2022 Sep 16;19(18):11711. doi: 10.3390/ijerph191811711 (PMC9517389; doi:10.3390/ijerph191811711)
Supplement: Supplementary file 1 [file ijerph-19-11711-s001.zip › ijerph-1860749-supplementary.pdf]

Table S1. Summary of Surface Swab Sample Results (pre and post-shooting).

Sample Number	Location Description	Pre Lead (µg/cm²)	Post Lead (µg/cm²)	Difference Lead (µg/cm²)	Acceptable Lead Loading
SW01	Range Officers Table - Centre of table	0.370	1.413	+1.043	0.5
SW02	Shooting Bench Lane 2 - Centre of bench	1.043	8.984	+7.941	0.5
SW03	Shooting Bench Lane 5 - Centre of bench	2.110	9.444	+7.334	0.5
SW04	Shooting Lane 7 - Floor in front of firing line	0.303	38.377	+38.074	0.1
SW05	Shooting Lane 9 - Floor in front of firing line	0.557	19.588	+19.031	0.1
SW06	Target Face 3 - Upper target	0.020	0.501	+0.481	0.5
SW07	Target Face 8 - Lower target	0.026	1.320	+1.294	0.5
SW08	Shooting Lane 4 - Top of upper target butt	1.019	1.502	+0.483	0.5
SW09	Shooting Lane 9 - Top of lower target butt	0.618	2.408	+1.79	0.5
